# Ginsenoside Rh2 Induces Human Hepatoma Cell Apoptosis via Bax/Bak Triggered Cytochrome C Release and Caspase-9/Caspase-8 Activation

**DOI:** 10.3390/ijms131215523

**Published:** 2012-11-22

**Authors:** Xiao-Xi Guo, Qiao Guo, Yang Li, Seung Ki Lee, Xue-Ning Wei, Ying-Hua Jin

**Affiliations:** 1 Key Laboratory for Molecular Enzymology and Engineering of the Ministry of Education, College of Life Science, Jilin University, Changchun 130012, China; E-Mails: gxxzmcn@me.com (X.-X.G.); guoqiao0808@163.com (Q.G.); liyang915@jlu.edu.cn (Y.L.); gloriashiningway@sohu.com (X.-N.W.); 2 College of Pharmacy, Seoul National University, Seoul 151-742, Korea; E-Mail: sklcrs@plaza.snu.ac.kr

**Keywords:** apoptosis, Bax/Bak translocation, caspase activation cascade, caspase-8/Bid pathway, ginsenoside Rh2

## Abstract

Ginsenoside Rh2 (G-Rh2) has been shown to induce apoptotic cell death in a variety of cancer cells. However, the details of the signal transduction cascade involved in G-Rh2-induced cell death is unclear. In this manuscript we elucidate the molecular mechanism of G-Rh2-induced apoptosis in human hepatoma SK-HEP-1 cells by demonstrating that G-Rh2 causes rapid and dramatic translocation of both Bak and Bax, which subsequently triggers mitochondrial cytochrome c release and consequent caspase activation. Interestingly, siRNA-based gene inactivation of caspase-8 effectively delays caspase-9 activation and apoptosis induced by G-Rh2, indicating that caspase-8 also plays an important role in the G-Rh2-induced apoptosis program. Taken together, our results indicate that G-Rh2 employs a multi pro-apoptotic pathway to execute cancer cell death, suggesting a potential role for G-Rh2 as a powerful chemotherapeutic agent.

## 1. Introduction

Ginseng, the root of *Panax ginseng* C.A. Meyer, is a medicinal plant used worldwide and has been reported to have various biological effects [1,2]. Ginsenosides are the major effective ingredients in ginseng [3–6]. Among them, ginsenoside Rh2 (G-Rh2) with a dammarane skeleton has been proven to have a remarkable potentiality of anti-proliferation [7–9] and pro-apoptosis [10–12]. In the human hepatoma cell line SK-HEP-1, low concentrations of G-Rh2 (1 μM) arrest the cell cycle at the G1/S transition phase by down-regulating the cyclin E-Cdk2 kinase activity [7]. At higher concentrations (12 μM), G-Rh2 induces acute and complete apoptotic cell death in a caspase-3 dependent but Bcl-2 independent manner [10]. In G-Rh2-induced cell apoptosis, p21*^WAF1/CIP1^* was shown to be cleaved by caspase 3. The truncation of p21*^WAF1/CIP1^* and consequent activation of cyclin A-Cdk2 kinase activity are prerequisite events for the execution of apoptosis induced by G-Rh2 [13]. However, the upper-stream signaling process of caspase-3 activation is unclear.

Apoptosis is an evolutionarily conserved form of cell suicide and requires a specialized proteolytic system involving a family of proteases called caspases. Two main caspase activation cascades have been described. One is initiated by the activation of cell-surface death receptors, such as Fas and tumor necrosis factor, leading to caspase-8 activation, which in turn cleaves and activates downstream effector caspases such as caspase-3, -6, and -7. An alternative mitochondrial pathway is triggered by cytochrome c released from mitochondria, which binds the caspase-activating protein Apaf-1, stimulating binding of Apaf-1 to pro-caspase 9 and inducing the processing and activation of this caspase [14,15]. The permeabilization of the mitochondrial outer membrane and cytochrome c release are regulated by Bcl-2 family proteins. The multi-domain pro-apoptotic molecules Bax and Bak serve as an obligatory gateway for cytochrome c release in response to diverse stimuli [16].

Deficiency in apoptosis may lead to tumor progression and resistance to chemotherapy [17]. Elucidating the molecular mechanisms associated with specific apoptotic processes, and thus triggering an effective apoptosis program, is a new therapeutic strategy to cure human cancers. The objective of this study was to examine the molecular mechanisms by which G-Rh2 induces acute and complete apoptosis in human cancer cells. In the present study, we show that G-Rh2 induces an immediate translocation of both Bax and Bak and consequent loss of mitochondrial membrane potential and cytochrome c release. Sequential activation of caspase-9, -3/-7 and -8 is involved in the G-Rh2-induced apoptosis program. In addition, caspase-9 can be independently activated by G-Rh2 in caspase-8-knockdowned SK-HEP-1 cells. Thus, G-Rh2 may be a promising anti-cancer reagent by triggering multiple apoptosis pathways.

## 2. Results

### 2.1. G-Rh2-Induced Apoptosis Is Mediated Via Caspase Activation

**Figure 1:**
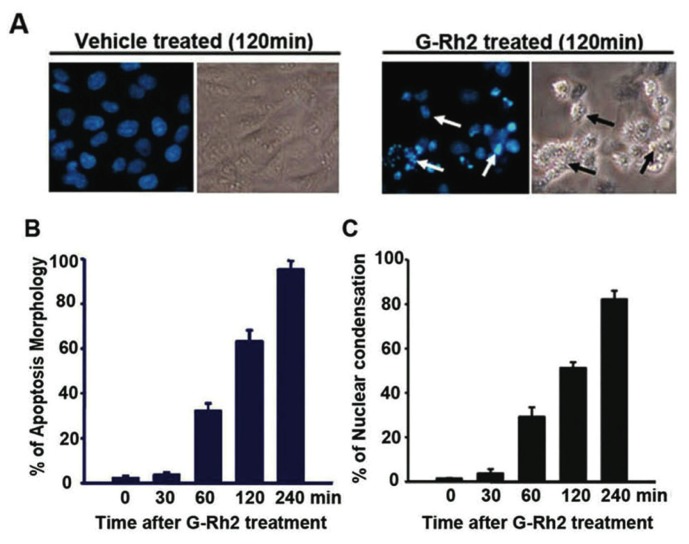

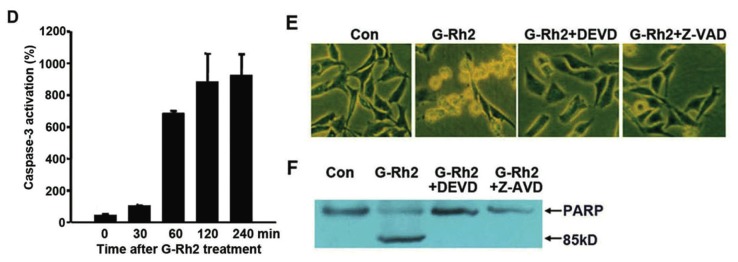
G-Rh2-induced SK-HEP-1 cell apoptosis is associated with increased caspase-3-like activity. (**A**) SK-HEP-1 cells were treated with 12 μM G-Rh2 for the indicated times, stained with 4′,6-diamidino-2-phenylindole (DAPI). The same region of cells was photographed by fluorescence microscopy (200×) under fluorescence and light fields. The white arrows and black arrows indicate the condensed or fragmented nuclear and multiblebbing cells, respectively; (**B**) The percentage of cells showing membrane blebbing; (**C**) The percentage of cells with condensed DNA; (**D**) Cell extracts were assayed for caspase-3 activity using fluoregenic caspase-3-specific substrate Ac-DEVD-AFC. Activation was measured as the increase in activity with respect to control cells (100%). Values were the mean ± SEM of five experiments. SK-HEP-1 cells were pretreated with DEVD-fmk or z-VYD-fmk for 30 min, and treated with 12 μM G-Rh2 for 120 min, and (**E**) photographed by phage-contrast microscopy; (**F**) cellular proteins were separated by sodium dodecyl sulfate polyacrylamide gel electrophoresis (SDS-PAGE) and immunoblotted for poly (ADP-ribose) polymerase (PARP).

It is known that G-Rh2 induces acute apoptotic cell death in human hepatoma SK-HEP-1 cells. In order to decipher the mechanism by which G-Rh2 triggers apoptosis in SK-HEP-1 cells, we examined the morphological and biochemical changes in cells upon G-Rh2 treatment. After treating SK-HEP-1 cells with G-Rh2 (12 μM) for 1 h, less than 5% scattered cells exhibited the typical apoptotic morphological changes, such as cell shrinkage, membrane blebbing, and nuclear condensation. Following 4 h of G-Rh2 treatment, over 85% of cells demonstrated characteristic apoptotic morphology (Figure 1A–C). DEVD-ase activity, indicating caspase-3 and -7 activities, was remarkably up-regulated in cell lysates by 60 min and increased over time (Figure 1D), and this up-regulation was consistent with the observed morphological changes. To further determine the involvement of caspases in G-Rh2-induced apoptosis, we employed a pharmacological inhibitor approach using the general caspase inhibitor Z-VAD-fmk and the caspase-3 and -7 inhibitor z-DEVD-fmk. Pre-treatment of cells with both inhibitors strongly inhibited G-Rh2-induced apoptosis, as evidenced by the examination of apoptotic morphology and poly (ADP-ribose) polymerase (PARP) cleavage (Figure 1E,F). These results demonstrate that G-Rh2 induces strong and acute apoptotic cell death in human hepatoma cells and caspases are intimately involved in this apoptosis.

### 2.2. G-Rh2 Induces Caspase-9,-3,-7, and -8 Cleavages in SK-HEP-1 Cells

**Figure 2:**
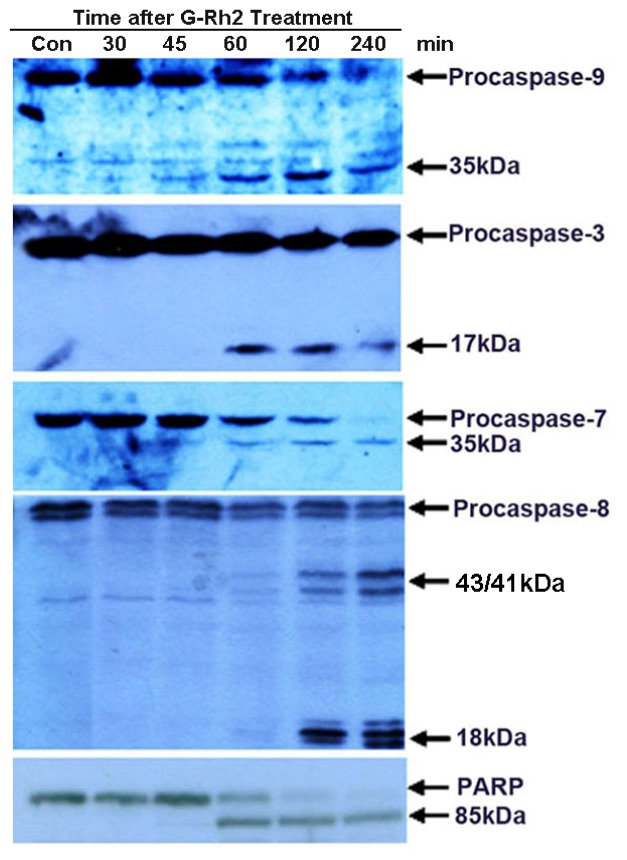
Sequential cleavage of caspase-9, -3/-7, and -8 during the apoptosis of SK-HEP-1 cells induced by G-Rh2. SK-HEP-1 cells were treated with 12 μM G-Rh2 for the indicated times, cell lysates were resolved by 12% SDS-PAGE and analyzed by immunobloting using specific antibodies to caspase-3, -7, -8, -9, and PARP. Procaspases and their cleaved forms are indicated by arrows.

Next we investigated the activation kinetics of specific caspases, namely caspase-8 and caspase-9, and the downstream effector caspases, caspase-3, -6, and -7. The results show that the initiator caspase-9, and the two effector caspases, caspase-3, -7, are detectably cleaved to yield catalytically active forms at 45 min and 60 min, after treatment with G-Rh2 (Figure 2). Caspase-6 was not detectable in SK-HEP-1 cells. The proteolytic cleavage of PARP was also detected at the same time point when caspase-3 and -7 were proteolytically cleaved (Figures 1F and 2). On the other hand, caspase-8, another initiator caspase, was proteolytically cleaved 120 min after G-Rh2 treatment (Figure 2). The activation of caspase-9, but not caspase-8, suggests that the pro-apoptotic effect of G-Rh2 may be elicited via both the mitochondrial and caspase-8 mediated pathways.

### 2.3. Apoptosis of G-Rh2-Induced SK-HEP-1 Cells Is Mediated through Cytochrome C Release and Depolarization of Mitochondrial Membrane Potential

**Figure 3:**
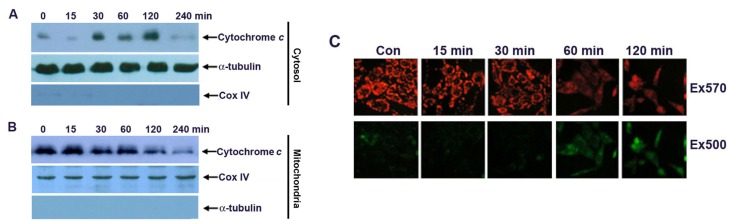
G-Rh2 induces rapid cytochrome c release and mitochondrial membrane depolarization in SK-HEP-1 cells. SK-HEP-1 cells were treated with 12 μM G-Rh2 for the indicated times. Equal amounts of cytosol (**A**) and mitochondrial (**B**) fractions of G-Rh2-treated cells were analyzed by immunoblotting for cytochrome c, α-tubulin, and Cox IV; (**C**) SK-HEP-1 cells were treated with 12 μM G-Rh2 for the indicated times, and stained with a mitochondria-specific cationic dye, MitoCapture, and photographed by fluorescence microscopy (200×) with excitation wavelengths of 570 nm (upper panel) and 500 nm (lower panel).

Cytochrome c release from the mitochondrial intermembrane space into the cytosol has been shown to be a key event in the activation of caspase-9, which subsequently initiates a caspase cascade involving caspase-3 and caspase-7 [14,18]. Since G-Rh2 induced the early activation of caspase-9, we then investigated cytochrome c release in G-Rh2 treated cells. Immunoblotting analysis demonstrated that cytochrome c in the cytoplasmic fraction appeared as early as 30 min and markedly increased at 120 min after G-Rh2 treatment (Figure 3A). In agreement with this, the protein levels of cytochrome c in the mitochondrial fraction were significantly decreased in a time-dependent manner (Figure 3B). The close association of cytochrome c release with subsequent activation of caspase 9 proves that G-Rh2 can induce apoptosis in SK-HEP-1 cells through the mitochondrial pathway.

Cytochrome c can be released from mitochondria into the cytosol through pores formed by the oligomerization of Bax and Bak on the outer mitochondrial membrane [19,20]. Therefore we investigated the mitochondrial membrane potential in G-Rh2 treated SK-HEP-1 cells. SK-HEP-1 cells were treated with G-Rh2 for the indicated times and stained with the cationic MitoCapture dye. This reagent is mitochondria-specific and forms aggregates in normally polarized mitochondria that exhibit bright red fluorescence after excitation at ~570 nm. In contrast, the monomeric form present in cells with depolarized mitochondrial membranes emits diffused green fluorescence after excitation at 500 nm. Our results show that the intensity of red fluorescence detectably decreases from 30 min and markedly diminishes 60 min after G-Rh2 treatment (Figure 3C, upper panel). In contrast, the intensity of green fluorescence increases inversely with decreasing red fluorescence in G-Rh2-induced cells (Figure 3C, lower panel). These results clearly suggest that G-Rh2-induced mitochondrial cytochrome c release is associated with the depolarization of the mitochondrial membrane potential.

### 2.4. G-Rh2-Induced Apoptosis in SK-HEP-1 Cells Is Associated with Mitochondrial Translocation of Bax and Bak

**Figure 4:**
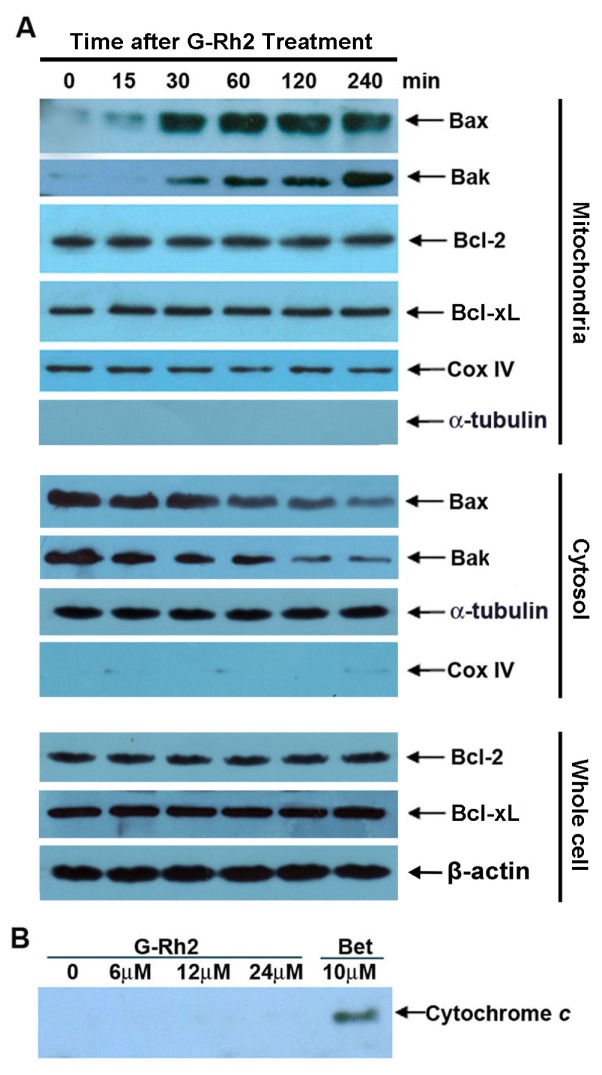
Both Bak and Bax rapidly and dramatically translocated to mitochondria in G-Rh2 treated SK-HEP-1 cells. (**A**) SK-HEP-1 cells were treated with 12 μM G-Rh2 for the indicated times. Equal amounts of whole-cell lysates, mitochondrial and cytosolic fractions were analyzed by immunoblotting for mitochondrial and cytosolic Bak, Bax, Cox IV and α-tubulin, cytosolic and whole-cell Bcl-2, Bcl-xL and whole-cell β-actin; (**B**) Isolated SK-HEP-1 cell mitochondria were treated with indicated concentration of G-Rh2 or betulinic acid (10 μM) for 60 min, and the supernatant were resolved by SDS-PAGE, and analyzed by immunoblotting using specific antibodies against cytochrome c.

The Bcl-2 family proteins regulate permeabilization of the mitochondrial outer membrane and therefore cytochrome c release, while the mitochondrial proteins Bax and Bak serve as a necessary gateway for cytochrome c release at the mitochondrial outer membrane [16]. We then determined the levels of Bax and Bak in mitochondrial fractions by immunoblotting analysis. As shown in Figure 4A, the levels of Bax and Bak in mitochondria were significantly increased as early as 30 min after G-Rh2 treatment, and this increase was in agreement with data showing the depolarization of the mitochondrial membrane potential and cytochrome c release (Figure 3A,C). At the same time, we determined the levels of two main anti-apoptosis Bcl-2 member proteins, Bcl-2 and Bcl-xL, in mitochondrial fractions and whole cell lysates. Importantly, the levels of both Bcl-2 and Bcl-xL on mitochondria stayed unchanged upon G-Rh2 treatment. In addition, the cytosol levels of Bak and Bax were both decreased upon G-Rh2 treatment (Figure 4A). These results indicated that G-Rh2 significantly promoted translocation of Bak and Bax from cytosol to mitochondria.

As G-Rh2 induces mitochondrial cytochrome c release within a short time after treatment with G-Rh2 in SK-HEP-1 cells, we asked the question whether G-Rh2 had a direct effect on the mitochondria membrane that enhances cytochrome c release. We then treated isolated mitochondria with increasing concentrations of G-Rh2 for 30 min and analyzed the mitochondrial supernatants for cytochrome *c*. In this study we used betulinic acid as a positive control, because previous studies had shown that this reagent directly induced cytochrome c release from isolated mitochondria [21]. As shown in Figure 4B, cytochrome c was not detected in the supernatant after incubating increasing concentrations (up to 24 μM) of G-Rh2 with isolated mitochondria. In contrast, betulinic acid (10 μM) treatment caused significant cytochrome c release.

Thus, we conclude that G-Rh2-induced mitochondria membrane deprivation and consequent cytochrome c release are most likely triggered by the increased mitochondrial Bax and/or Bak.

### 2.5. Caspase-8 Is Also Activated in G-Rh2-Treated Cells

**Figure 5:**
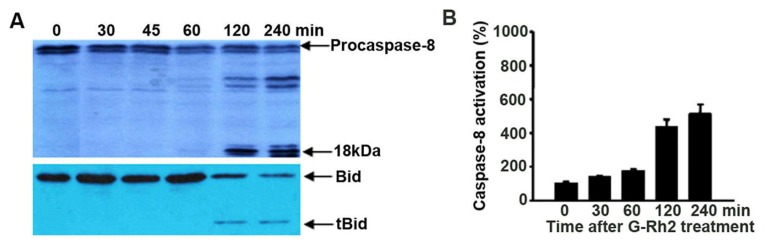
Kinetics of caspase-8 processing, Bid cleavage, and activation of caspase-8 following G-Rh2 treated SK-HEP-1 cells. (**A**) Processing of caspase-8 and cleavage of Bid were determined by immunoblotting analysis; (**B**) Cell-free caspase-8 activities were analyzed by using specific substrates, Ac-IETD-AFC. Activation was measured as the increase in activity with respect to control cells (100%). Values were the mean ± SEM of five experiments.

It has been well documented that caspase-8 is the first caspase activated in membrane receptor (such as Fas, tumor necrosis factor-related apoptosis-inducing ligand (TRAIL), and tumor necrosis factor (TNF))-mediated apoptosis [22]. We also examined whether caspase-8 activation was promoted by G-Rh2. Caspase-8 cleavage was assessed by Western blotting, and caspase-8 activity was analyzed by a cell-free protease assay that measures the cleavage activity of caspase-8-specific fluorescence labeled Ac-IETD-AFC. Caspase-8 cleavage occurred at 60–90 min after G-Rh2 treatment (Figures 2 and 5A,B), indicating that caspase-8 activation is also a pivotal event in G-Rh2-induced apoptosis.

Activated caspase-8 may further enhance apoptosis by cleaving the pro-apoptotic protein Bid [22]. Cleaved Bid binds to mitochondria, antagonizes the anti-apoptotic Bcl-2 family proteins and causes a further efflux of cytochrome c into the cytosol [23]. Moreover, Bid is also the substrate of casaspe-3, Bid cleavage is another hallmark for caspase-3 activation [24]. Therefore, we investigated whether G-Rh2 elicited the cleavage of Bid. The results showed that a significant decrease of uncleaved Bid and the occurrence of tBid were detected in cells after 120 min of treatment with G-Rh2, when caspase-8 was activated (Figure 5A,B). This observation implies that G-Rh2 provokes the apoptosis of SK-HEP-1 cells by activating both caspase-8 and caspase-9.

**Figure 6:**
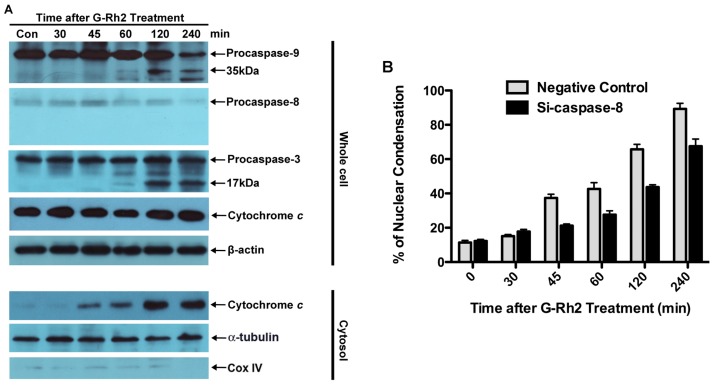
Caspase-8 siRNA notably delayed G-Rh2-induced caspase-9 and -3 activation, partially attenuated cytochrome c release and consequent cell apoptosis. SK-HEP-1 cells were transiently transfected with either negative control siRNA or siRNA against caspase-8 and treated with 12 μM G-Rh2 for indicated times. (**A**) The cleavage of caspase-8, -9 and -3 in whole-cell lysates and cytochrome c release in the cytosol were examined by immunoblotting; (**B**) The nuclear condensation of cells undergoing apoptosis was measured by DAPI staining.

To provide more evidence for this notion, we examined the contribution of caspase-8 to G-Rh2-induced apoptosis by knocking down caspase-8 expression with small interfering RNA. As shown in Figure 6, G-Rh2-induced caspase-9 and -3 cleavage and cell nuclear condensation were remarkably delayed by at least 60 min by the knockdown of caspase-8. However the release of cytochrome c was just partially attenuated by caspase-8 knockdown. In contrast, G-Rh2-promoted caspase-8, -9 and -3 cleavage, and cytochrome c release was not influenced in cells transfected with non-targeting negative control siRNA (data not shown). These results suggest that caspase-8 and -9 both play important roles in cell death induced by G-Rh2. The cleavage of Bid by caspase-8 may reinforce mitochondria-mediated apoptotic pathways, but is not essential for caspase-9 activation.

## 3. Experimental Section

### 3.1. Materials

Ginsenoside Rh2 was dissolved in 75% ethanol at a concentration of 12 mM and stored at −20 °C. Dulbecco’s modified eagle’s medium (DMEM) and calf serum were obtained from Gibco BRL. Mouse anti-caspase-3 antibody was purchased from Cell Signaling Technology (Beverly, MA, USA), mouse anti-cytochrome c and caspase-6, -7, -9 antibodies were purchased from BD Pharmingen™ (Franklin Lakes, NJ, USA). Goat anti-caspase-8 and rabbit anti-PARP antibodies were from Santa Cruz Biotechnology (Santa Cruz, CA, USA). Caspase substrates Ac-DEVD-AFC, Ac-IETD-AFC, Ac-LEHD-AFC were from BD Pharmingen™. The Mitochondria Isolation Kit was obtained from PIERCE (Rockford, IL, USA). All other drugs and chemicals were from Sigma (St. Louis, MO, USA).

### 3.2. Cell Culture Conditions and Apoptosis Assays

SK-HEP-1 cells were maintained as a monolayer culture in DMEM supplemented with 5% (by volume) heat inactivated calf serum, 100 U/mL penicillin, 100 μg/mL streptomycin. The cells were treated with G-Rh2 in serum-free DMEM for the indicated time. Both floating and adherent cells were harvested for immunoblotting and caspase activity assays. At the same time, another set of G-Rh2 treated cells were fixed and permeabilized in 4% paraformaldehyde and then stained with DAPI solution to visualize the nuclei and DNA. Fluorescent cells were photographed under the fluorescence microscope.

### 3.3. Immuoblot Analysis

Cells were washed with ice-cold phosphate buffered saline (PBS) and solublized in a lysis buffer containing 20 mM Tris, pH 7.5, 0.5% Triton X-100, 2 mM MgCl_2_, 1 mM DTT, 1 mM EGTA, 50 mM β-glycerol phosphate, 25 mM NaF, 1 mM Na_3_VO_4_, 2 μg/mL leupeptin, 2 μg/mL pepstatin A, 2 μg/mL antipain and 1 mM PMSF. After incubation on ice for 1 h, the insoluble materials were removed by centrifugation at 12,000 rpm for 15 min. Fifty micrograms of protein from each sample were analyzed by SDS-polyacrylamide gel electrophoresis (PAGE) followed by electrotransfer onto polyvinylidene difluoride (PVDF) membrane (Gelman). Membranes were blocked with 5% nonfat milk and probed with the indicated antibodies. The blots were washed and incubated with a horseradish peroxidase-coupled anti mouse IgG or anti rabbit IgG antibody (Pierce, Rockford, IL, USA) followed by detection with ECL revelation system (Amersham, Piscataway, NJ, USA).

### 3.4. Caspase Assay

Fifty micrograms of cell lysates were incubated with 200 nM Ac-DEVD-AFC (for caspase-3), Ac-IETD-AFC (for caspase-8), Ac-LEHD-AFC (for caspase-9) in reaction buffer (20 mM Hepes pH 7.4, 100 mM NaCl, 10 mM DTT, 0.1% CHAPS, 10% Sucrose) at 37 °C for 1 h. The reaction was monitored by fluorescence emission at 505 nm (excitation at 405 nm)

### 3.5. Preparation of Mitochondrial and Cytosolic Protein Extracts and Cytochrome C Release Assay

Mitochondria and cytosolic protein extracts were prepared using a Mitochondria Isolation Kit according to the manufacturer’s instructions. Isolated mitochondria were solublized in a lysis buffer containing 20 mM Tris, pH 7.5, 150 mM NaCl, 1% NP-40, 0.5% Deoxycholate, 0.1% SDS, 2 mM MgCl_2_, 1 mM DTT, 1 mM EGTA, 50 mM β-glycerol phosphate, 25 mM NaF, 1 mM Na_3_VO_4_, 2 μg/mL leupeptin, 2 μg/mL pepstatin A, 2 μg/mL antipain and 1 mM PMSF. The mitochondria and cytosol proteins were subjected to immunoblotting using mouse monoclonal anticytochrome c antibodies.

### 3.6. Depolarization Assay of Mitochondrial Membrane Potential by Staining with MitoCapture

Cells were incubated with 1 μg/mL of MitoCapture cation dye (Calbiochem, La Jolla, CA, USA) at 37 °C for 30 min. Cells were photographed using a fluorescence microscope (Olympus, Tokyo, Japan) with excitation wavelengths of 500 and 570 nm.

### 3.7. RNA Interference

siRNA duplexes for caspase-8 and negative controlled non-targeting siRNA were purchased from Bioneer (Daejeon, Korea). The sequences of siRNA duplexes are listed in Table 1. A total of 3 × 10^5^ SK-HEP-1 cells in six-well dishes were transfected with a final concentration of 100 nM siRNA duplexes using HiPerFect Transfection Reagent (Qiagen, Valencia, CA, USA), according to the manufacturer’s instructions. After incubation for 24 h, cells were treated with G-Rh2 for 30 min, 45 min, 60 min, 120 min or 240 min, respectively. Finally, the total proteins in the cell lysates were prepared for the caspase-9 activity assay and Western blot analysis of caspase-8.

## 4. Discussion

An increasing number of studies have implicated apoptosis as an important mechanism by which chemotherapeutic agents kill susceptible cells [25]. The present study and previous reports have demonstrated that G-Rh2 is able to induce apoptosis in different strains of tumor cells of human [10–12] and murine origin. Some of the molecular and biochemical pathways involved in G-Rh2-induced apoptosis were investigated in human cancer cells [13,26–28]. Moreover, it has been reported that the over-expression of Bcl-2 or Bcl-xL does not suppress G-Rh2-induced apoptosis [10,11], which implies that G-Rh2 promoted cell death is not merely mediated via the mitochondrial pathway.

Here, we have proven that G-Rh2 induces apoptosis by a mechanism that is functionally associated with depolarization of the mitochondrial membrane potential and caspase-8 activation. G-Rh2-induced apoptosis involves sequential proteolytic activation of caspase-9, which is detected 45 min after treatment, and caspases-3 and -7, which are detectable 60 min after treatment (Figure 2). These time courses are consistent with those of caspase-3 activation and PARP cleavage (Figures 1 and 2). As expected, the activation kinetics of the initiator caspase-9 and of the executioner caspase-3 correlate well in a time-dependent fashion with those of the mitochondrial membrane depolarization and subsequent release of cytochrome c from mitochondria (Figures 3A,B). On the other hand, the activation of caspase-8, the initiator caspase of the extrinsic pathway, only became evident later at 120 min after treatment (Figures 2, 5 and 6). This finding suggests that caspase-9 and caspase-8 may both be responsible for the cleavage and activation of caspase-3. In agreement with this hypothesis, we found that when caspase-8 became activated after 120 min of G-Rh2 treatment, an obvious caspase-8-dependent Bid cleavage occurred in SK-HEP-1 cells (Figure 5). Furthermore, suppression of caspase-8 expression by siRNA temporally delayed caspase-9 activation and apoptosis (Figure 6). These results clearly suggest that the activation of caspase-8, although later than caspase-9 activation, could be an alternative mechanism for G-Rh2-induced apoptosis.

Our present results may help to interpret previous reports where over-expression of Bcl-2 or Bcl-XL did not reduce the extent of apoptosis induced by G-Rh2. Here we show that G-Rh2 induces rapid and dramatic mitochondrial translocation of both Bak and Bax. In contrast, the levels of mitochondria-associated Bcl-2 and Bcl-XL were not altered upon G-Rh2 treatment (Figure 4A). Therefore, the translocation of Bak and Bax to the mitochondria and their consequent pro-apoptotic activity may largely overwhelm the anti-apoptotic capacity of Bcl-2 and Bcl-XL in G-Rh2-induced apoptosis programs. Moreover, the activation of caspase-8 also plays an important role in G-Rh2-induced SK-HEP-1 cell apoptosis (Figures 2, and 5A,B). Caspase-8 initiated by the caspase-activation cascade could bypass the anti-apoptotic effects of Bcl-2 and Bcl-XL [22]. Bcl-2 and Bcl-XL are frequently over-expressed in many cancers and contribute to tumor initiation, progression and resistance to therapy [29]. Because of those distinct advantages, G-Rh2 may have significant importance as an application in anticancer therapy, especially in combination treatment.

## 5. Conclusions

In conclusion, G-Rh2-induced apoptosis is associated with the early activation of caspase-9 through the mitochondrial pathway, as demonstrated by the early loss of mitochondrial membrane potential and cytochrome c release from mitochondria. In particular, both Bax and Bak are rapidly and dramatically translocated to the mitochondria in G-Rh2 treated cells without additional expression of either protein. The activation of caspase-8 and Bid cleavage is also triggered by G-Rh2. These results, together with previous findings, evidently show the multipath pro-apoptogenic effects of G-Rh2 on tumor cells, suggesting a potential role of G-Rh2 as a powerful chemotherapeutic agent.

## Figures and Tables

**Table 1:** siRNA duplexes used for caspase-8 knockdown.

Sequence number	siRNA Duplex	

	Sense (5′-3′)	Antisense (5′-3′)
1	GCUGCUCUUCCGAAUUAAU	AUUAAUUCGGAAGAGCAGC
2	CCUGCUGGAUAUUUUCAUA	UAUGAAAAUAUCCAGCAGG
3	CAGAUCAGAAUUGAGGUCU	AGACCUCAAUUCUGAUCUG
Negative control	CCUACGCCACCAAUUUCGU	ACGAAAUUGGUGGCGUAGG

## References

[1] Park H.M., Kim S.J., Kim J.S., Kang H.S. (2012). Reactive oxygen species mediated ginsenoside Rg3- and Rh2-induced apoptosis in hepatoma cells through mitochondrial signaling pathways. Food Chem. Toxicol.

[2] Chan S.W. (2012). *Panax ginseng*, *Rhodiola rosea* and *Schisandra chinensis*. Int. J. Food Sci. Nutr.

[3] Jin Y.H., Yim H., Park J.H., Lee S.K. (2003). Cdk2 activity is associated with depolarization of mitochondrial membrane potential during apoptosis. Biochem. Biophys. Res. Commun.

[4] Kaneko H., Nakanishi K. (2004). Proof of the mysterious efficacy of ginseng: Basic and clinical trials: Clinical effects of medical ginseng, korean red ginseng: Specifically, its anti-stress action for prevention of disease. J. Pharmacol. Sci.

[5] Cheng Y., Shen L.H., Zhang J.T. (2005). Anti-amnestic and anti-aging effects of ginsenoside Rg1 and Rb1 and its mechanism of action. Acta Pharm. Sinica.

[6] Nam M.H., Kim S.I., Liu J.R., Yang D.C., Lim Y.P., Kwon K.H., Yoo J.S., Park Y.M. (2005). Proteomic analysis of Korean ginseng (*Panax ginseng* C.A. Meyer). J. Chromatogr. B.

[7] Lee K.Y., Park J.A., Chung E., Lee Y.H., Kim S.I., Lee S.K. (1996). Ginsenoside-Rh2 blocks the cell cycle of SK-HEP-1 cells at the G1/S boundary by selectively inducing the protein expression of p27kip1. Cancer Lett.

[8] Oh M., Choi Y.H., Choi S., Chung H., Kim K., Kim S.I., Kim D.K., Kim N.D. (1999). Anti-proliferating effects of ginsenoside Rh2 on MCF-7 human breast cancer cells. Int. J. Oncol.

[9] Li B., Zhao J., Wang C.Z., Searle J., He T.C., Yuan C.S., Du W. (2011). Ginsenoside Rh2 induces apoptosis and paraptosis-like cell death in colorectal cancer cells through activation of p53. Cancer Lett.

[10] Park J.A., Lee K.Y., Oh Y.J., Kim K.W., Lee S.K. (1997). Activation of caspase-3 protease via a Bcl-2-insensitive pathway during the process of ginsenoside Rh2-induced apoptosis. Cancer Lett.

[11] Kim H.E., Oh J.H., Lee S.K., Oh Y.J. (1999). Ginsenoside RH-2 induces apoptotic cell death in rat C6 glioma via a reactive oxygen- and caspase-dependent but Bcl-X(L)-independent pathway. Life Sci..

[12] Fei X.F., Wang B.X., Tashiro S., Li T.J., Ma J.S., Ikejima T. (2002). Apoptotic effects of ginsenoside Rh2 on human malignant melanoma A375-S2 cells. Acta Pharmacol. Sinica.

[13] Jin Y.H., Yoo K.J., Lee Y.H., Lee S.K. (2000). Caspase 3-mediated cleavage of p21WAF1/CIP1 associated with the cyclin A-cyclin-dependent kinase 2 complex is a prerequisite for apoptosis in SK-HEP-1 cells. J. Biol. Chem.

[14] Shi Y. (2002). Mechanisms of caspase activation and inhibition during apoptosis. Mol. Cell.

[15] Wurstle M.L., Laussmann M.A., Rehm M. (2012). The central role of initiator caspase-9 in apoptosis signal transduction and the regulation of its activation and activity on the apoptosome. Exp. Cell Res.

[16] Wei M.C., Zong W.X., Cheng E.H., Lindsten T., Panoutsakopoulou V., Ross A.J., Roth K.A., MacGregor G.R., Thompson C.B., Korsmeyer S.J. (2001). Proapoptotic BAX and BAK: A requisite gateway to mitochondrial dysfunction and death. Science.

[17] Brown J.M., Attardi L.D. (2005). The role of apoptosis in cancer development and treatment response. Nat. Rev. Cancer.

[18] Hengartner M.O. (2000). The biochemistry of apoptosis. Nature.

[19] Ricci J.E., Munoz-Pinedo C., Fitzgerald P., Bailly-Maitre B., Perkins G.A., Yadava N., Scheffler I.E., Ellisman M.H., Green D.R. (2004). Disruption of mitochondrial function during apoptosis is mediated by caspase cleavage of the p75 subunit of complex I of the electron transport chain. Cell.

[20] Dewson G., Kluck R.M. (2009). Mechanisms by which Bak and Bax permeabilise mitochondria during apoptosis. J. Cell Sci.

[21] Fulda S., Scaffidi C., Susin S.A., Krammer P.H., Kroemer G., Peter M.E., Debatin K.M. (1998). Activation of mitochondria and release of mitochondrial apoptogenic factors by betulinic acid. J. Biol. Chem.

[22] Thorburn A. (2004). Death receptor-induced cell killing. Cell. Signal.

[23] Luo X., Budihardjo I., Zou H., Slaughter C., Wang X. (1998). Bid, a Bcl2 interacting protein, mediates cytochrome c release from mitochondria in response to activation of cell surface death receptors. Cell.

[24] Degli Esposti M., Ferry G., Masdehors P., Boutin J.A., Hickman J.A., Dive C. (2003). Post-translational modification of Bid has differential effects on its susceptibility to cleavage by caspase 8 or caspase 3. J. Biol. Chem.

[25] Johnstone R.W., Ruefli A.A., Lowe S.W. (2002). Apoptosis: A link between cancer genetics and chemotherapy. Cell.

[26] Ham Y.M., Choi J.S., Chun K.H., Joo S.H., Lee S.K. (2003). The c-Jun *N*-terminal kinase 1 activity is differentially regulated by specific mechanisms during apoptosis. J. Biol. Chem.

[27] Ham Y.M., Lim J.H., Na H.K., Choi J.S., Park B.D., Yim H., Lee S.K. (2006). Ginsenoside-Rh2-induced mitochondrial depolarization and apoptosis are associated with reactive oxygen species- and Ca^2+^-mediated c-Jun NH_2_-terminal kinase 1 activation in HeLa cells. J. Pharmacol. Exp. Therapeut.

[28] Oh J.I., Chun K.H., Joo S.H., Oh Y.T., Lee S.K. (2005). Caspase-3-dependent protein kinase C delta activity is required for the progression of Ginsenoside-Rh2-induced apoptosis in SK-HEP-1 cells. Cancer Lett.

[29] Kirkin V., Joos S., Zornig M. (2004). The role of Bcl-2 family members in tumorigenesis. Biochim. Biophys. Acta.

